# Severe viral infections requiring intensive care unit admissions- aetiology, co-infections, respiratory interventions and outcomes

**DOI:** 10.1007/s15010-025-02637-2

**Published:** 2025-09-09

**Authors:** M Brown, F Abeer, T Roe, R Beecham, O Arscott, B Eastwood, S Mahar, M Montague, D Neseam, P Patel, J Srinivasa, A Greenwell, K Thomas, D Browning, E Wilson-Davies, A Conway Morris, MPW Grocott, K Saeed, A Dushianthan

**Affiliations:** 1 General Intensive Care Unit, University Hospital Southampton NHS Foundation Trust, Tremona Road, Southampton, SO16 6YD UK; 2 Microbiology Department, University Hospital Southampton NHS Foundation Trust, Tremona Road, Southampton, SO16 6YD UK; 3 Southampton Specialist Virology Centre, University Hospital Southampton NHS Foundation Trust, Tremona Road, Southampton, SO16 6YD UK; 4 Department of Medicine, Addenbrooke’s Hospital, University Division of Anaesthesia, University of Cambridge, Hills Road, Cambridge, CB2 0QQ UK; 5 Integrative Physiology and Critical Illness Group, Clinical and Experimental Sciences, Faculty of Medicine, University of Southampton, Southampton, SO16 6YD UK; 6 Perioperative and Critical Care theme, NIHR Southampton Biomedical Research Centre, University Hospital Southampton / University of Southampton, Southampton, SO16 6YD UK

**Keywords:** Pneumonia, Viral infection, Intensive care, Mechanical ventilation

## Abstract

**Introduction:**

Severe viral infections are common in patients requiring admission to intensive care units (ICU). Furthermore, these patients often have additional secondary or co-infections. Despite their prevalence, it remains uncertain to what extent those additional infections contribute to worse outcomes for patients with severe viral infections requiring ICU admission. This study aims to characterise severe viral infections requiring admission to intensive care, and describe their viral aetiology, the incidence of additional infections, and their clinical outcomes.

**Methods:**

This retrospective single-centre cohort included consecutive adults admitted to the intensive care unit (ICU) with a positive polymerase chain reaction (PCR) test for viral infection from 2015 to 2024. Patients with SARS-CoV-2 were not included in this analysis. The data were retrieved from all available electronic databases. Patients were further stratified to compare severe viral infections alone to those with other microbiology confirmed co-infection (within 48 h of admission) and secondary infection (48 h after ICU admission).

**Results:**

We identified 222 with positive PCR for viral infection admitted to ICU. The majority were admitted with radiographic evidence of pneumonia (73.0%). Rhinovirus (28.4%), influenza A (18.5%), and RSV (16.2%) were the most common viral pathogens. Of the total, 149 patients had viral infection alone, 50 had co-infections, and 23 developed secondary infections. 30-day and ICU mortality were similar for viral alone, co-infection and secondary infection groups. Although those with secondary infection had a greater hospital and ICU length of stay, this was not reflected in the duration of mechanical ventilation or 30-day hospital mortality.

**Conclusion:**

In our large cohort of severe viral infections where Rhinovirus was the most common pathogen. This patient population constitute a high burden of respiratory support. The study also characterised 22.5% had co-infection, and 10% had subsequent secondary infection. While patients with secondary infections had prolonged ICU and hospital stay, the 30-day mortality was similar between all groups.

**Supplementary Information:**

The online version contains supplementary material available at 10.1007/s15010-025-02637-2.

## Introduction

Respiratory failure from severe infections is one of the leading causes of intensive care unit (ICU) admissions worldwide. Severe viral infections are commonly identified, and the incidence of viral pneumonia have increased over the recent years [1]. Moreover, the development of new diagnostic methods, including direct antigen tests, real time multiplex polymerase chain reaction (PCR), rapid and point of care diagnostic PCRs and more recently metagenomic sequencing as led to increased detection of respiratory pathogens in patients admitted to critical care for respiratory support [2, 3]. Patients admitted to the ICU often present with radiographic features of pneumonia and require non-invasive and/or invasive advanced respiratory support for acute hypoxemic respiratory failure [4, 5]. In addition to the recent COVID-19 pandemic, other seasonal and non-seasonal viruses such as influenza A and B, rhinovirus, parainfluenza and respiratory syncytial viruses (RSV) are frequently detected in patients on intensive care with acute respiratory failure [6]. Understanding the exact proportion of critical care admissions due to viral infections is challenging. Incidences vary seasonally and establishing causation between viral detection and clinical illness is not always straightforward. Prior to the COVID-19 pandemic, the reported proportion of patients with viral pneumonia requiring admission to critical care varied between 16% and 49% [7–9]. Despite this common occurrence, the management strategies and outcomes of severe respiratory viral infections (with no identified additional bacterial or fungal co-pathogens) leading to critically illness have likely been insufficiently studied.

Viral-bacterial or viral-other pathogens co- or secondary infections pose a potentially serious risk to ICU patients, where such viral-bacterial interactions have been shown to enhance bacterial adherence to epithelial surfaces, dysregulation of innate immune responses, and reduced pathogen clearance leading to potentially adverse clinical outcomes [10]. A meta-analysis by Burk et al. (2016), found an increased mortality rate amongst viral-bacterial co-infection patients compared to those with viral infection alone [11]. Secondary bacterial infection is also common after a severe viral infection due to impaired immunity and alterations in lung microbiome [12, 13]. Viral-bacterial co-infections or secondary infections may contribute to worse outcomes in critically ill ICU patients [14–16]. Moreover, it is often difficult to decipher if the clinical deterioration is primarily related to viral infection, other pathogen co-infection or a combination of both. In this study, we aimed to describe the aetiology, respiratory interventions provided, and outcomes of patients admitted to ICU with severe respiratory viral infection. We also aimed to describe in detail the nature of co-infections and secondary infections in relation to ICU interventions and outcomes.

## Methods

### Study population

This is a single-centre retrospective observational study of all adults (age ≥ 18 years old) admitted to ICU with PCR-confirmed (non-COVID-19) viral infections between 01/2015 and 06/2024. The study formed part of a large cohort study (CRIT-CO) investigating outcomes for critically ill patients in our ICU. The study was sponsored by the University Hospital Southampton NHS Foundation Trust (RHM CRI 0370) and approved by the Health Research Authority and Health and Care Research Wales (HCRW) (IRAS 232922, approval date: 26/11/2018). All identifiable patient data has been anonymised, and, due to the retrospective observational nature of the study, consent was waived. This study is compliant with Health Research Authority (HRA) ethical standards.

### Data collection

The ICU and hospital clinical notes system (MetaVision (iMDsoft, Tel Aviv, Israel)) and CHARTS (custom software for University Hospital Southampton NHS Trust, version 35)) were reviewed allowing for the extraction of all relevant information. Most quantitative data was extracted automatically, however further manual data extraction was required for confirmation and quality assurance. Hospital and ICU admission dates, admission diagnosis, past medical history, drug history, social history, and clinical, biochemical and microbiological data were extracted using these platforms.

Past medical history including presence of asthma, chronic obstructive pulmonary disease (COPD), bronchiectasis, interstitial lung disease, hypertension, ischaemic heart disease, myocardial infarction, chronic kidney disease, liver cirrhosis, and cancer (of any aetiology, within 5 years, not necessarily receiving active treatment) were extracted. Charlson’s comorbidity index was calculated to facilitate the degree of disease burden from chronic illnesses [17]. Medications prior to admission were also screened, including inhaler use, chronic oral steroid use (daily or more than 4 courses within /year), and immunosuppressant medications. We identified active smokers but could not define with certainty the pack-year history.

We reported the mode of oxygen therapy delivery (including facemask, high flow nasal oxygen (HFNO), continuous positive airway pressure (CPAP), non-invasive ventilation (NIV) and invasive mechanical ventilation (IMV). The use of HFNO and NIV was also reported cumulatively as non-invasive respiratory support (NIRS). Therapeutic failure of each type of oxygen therapy delivery support, defined as escalation to a more invasive form, or death. For each patient, microbiological data including blood culture, sputum culture, nasopharyngeal swabs, and endotracheal aspirate (ETA) cultures, small volume bronchoalveolar lavage (BALF) cultures, and urinary antigen to pneumococcal (*Streptococcus pneumoniae)* and legionella (*Legionella pneumophila*) were collected. The Multiplex assay panel PCR with primers obtained from Integrated DNA technologies (IDT) USA Applied Biosystems (ThermoFisher Scientific, Waltham, MA, USA). All viral positive PCR were included regardless of if they were obtained from nasopharyngeal swabs, sputum, ETA or BALF samples. All patients had routine hourly observations and daily bloods including full blood count and C-reactive protein. Daily laboratory inflammatory panels (CRP, neutrophils, lymphocytes and platelets) up to day 5 after ICU admission were also captured.

### Definitions

For the admission diagnosis of pneumonia, we adopted the British Thoracic Society guidelines: clinical signs consistent with an acute lower respiratory tract infection together with new radiographic features, for which there is no other explanation, and as the primary reason for hospital admission and is managed as pneumonia [18]. Multi-organ dysfunction was defined as the development of physiological derangement of two or more organ systems using biochemical, or clinical parameters. COPD and asthma exacerbations defined using NICE guidelines [19, 20].

### Exposure groups

Positive virology and microbiology results were screened by a consultant microbiologist/ICU infection specialist to determine the clinical significance of the result. All Patients with severe viral infection were further stratified by viral alone (single exclusive) or patients with co-infection defined as the presence of 1 or more significant microbiological results isolated during admission or secondary infection, defined as those with a significant microbiological result isolated 48 h to seven days from admission with a severe viral infection.

### Outcomes

The primary outcome reported was 30-day hospital mortality. Secondary outcomes were ICU mortality, length of hospital stay, length of ICU stay, duration of mechanical ventilation and requirement of invasive mechanical ventilation in survivors.

### Statistical analysis

We tested for normality using the Shapiro–Wilks test, and as our dataset was non-normally distributed, continuous variables were reported as median with inter-quartile range (IQR). Baseline characteristics are described by median with IQR for continuous variables and counts with percentages for categorical variables. Kruskal–Wallis’s test and Fischer’s exact test for continuous and binary outcomes, respectively. Kaplan-Meier and Cox proportional hazards models were constructed for analysis of hospital and ICU length of stays, which were defined as the time elapsed between admission and death or censorship, whichever occurred first. Adjustment was made for a priori selection of common confounders such as age and comorbidity. All model assumptions were tested graphically. All analyses were performed using R (version 4.2.2).

## Results

### Patient demographics and baseline characteristics

During this study period, 222 patients admitted to the ICU with positive viral PCR (non-COVID-19) with a median age of 66.1 years (IQR: 49.8–75.2), predominantly male (54.5%) and of white ethnicity (82.0%). Comorbidities across the cohort included chronic respiratory conditions (41.4%) including asthma (18.5%) and COPD (19.8%). A diagnosis of cancer (active or < 5 years) was also common (23.0%). Admission medications include inhaled corticosteroids (18.9%), oral corticosteroids (22.1%) and other immunosuppressive drugs (12.2%). Reasons for admission featured respiratory compromise including pneumonia (73.0%). Detailed description of demographics is presented in Table 1. For those without a diagnosis of pneumonia on admission (Supplementary table S1), reasons for admission included potential or actual involvement of the respiratory system including multi-organ dysfunction (28.3%), or exacerbations of existing respiratory conditions such COPD (21.7%) or asthma (13.3%).

**Table 1:** Baseline characteristics of patients with severe viral infections admitted to intensive care and further stratified between those who survived or died at 30 days. Abbreviations: BMI, body mass index; COPD, chronic obstructive pulmonary disease; IHD, ischaemic heart disease.^1^ Mann-Whiney U test and fischer’s exact test

Characteristics	All	Survived	Died	*p*-value^1^
Number of patients	222	168	54	
Age	66.1 (49.8–75.2)	63.9 (46.5–74.6)	71.5 (59.8–76.1)	0.02
Male	121 (54.5%)	89 (53.0%)	32 (59.3%)	0.42
Ethnicity				
Asian or Asian British	8 (3.6%)	8 (4.8%)	0 (0.0%)	**0.01**
Black or black British	2 (0.9%)	0 (0.0%)	2 (3.7%)	
White	182 (82.0%)	140 (83.3%)	42 (77.8%)	
Other ethnicity group	3 (1.3%)	1 (0.6%)	2 (3.7%)	
Unknown	27 (12.2%)	19 (11.3%)	8 (14.8%)	
BMI	26.6 (23.2–31.5)	26.4 (23.1–31.6)	27.5 (24.2–31.2)	0.60
**Comorbidities**				
Charlson comorbidity index	3.0 (1.0, 5.0)	3.0 (1.0, 4.0)	5.0 (3.0, 6.0)	**< 0.01**
Chronic respiratory condition	92 (41.4%)	70 (41.7%)	22 (40.7%)	0.90
Asthma	41 (18.5%)	38 (22.6%)	3 (5.6%)	**0.01**
COPD	44 (19.8%)	27 (16.1%)	17 (31.5%)	**0.01**
Bronchiectasis	4 (1.8%)	0 (0.0%)	4 (7.4%)	**< 0.01**
Interstitial lung disease	6 (2.7%)	4 (2.4%)	2 (3.7%)	0.63
IHD	22 (9.9%)	16 (9.5%)	6 (11.1%)	0.73
Hypertension	70 (31.5%)	47 (28.0%)	23 (42.6%)	**0.04**
Myocardial infarction	15 (6.8%)	11 (6.5%)	4 (7.4%)	0.76
Chronic kidney disease	21 (9.5%)	15 (8.9%)	6 (11.1%)	0.63
Liver cirrhosis	8 (3.6%)	6 (3.6%)	2 (3.7%)	1.0
Cancer	51 (23.0%)	33 (19.6%)	18 (33.3%)	**0.04**
Smoker	69 (31.1%)	53 (31.5%)	16 (29.6%)	0.79
**Medications prior to admission**				
Inhaled steroids	42 (18.9%)	34 (20.2%)	8 (14.8%)	0.38
Immunosuppressive drugs	27 (12.2%)	14 (8.3%)	13 (24.1%)	**0.02**
Oral steroids	49 (22.1%)	36 (21.4%)	13 (24.1%)	0.79
**Reason for admission**				
Pneumonia	162 (73.0%)	121 (72.0%)	41 (75.9%)	0.57
Multi-organ dysfunction	71 (32.0%)	49 (29.2%)	22 (40.7%)	0.11
COPD exacerbation	31 (14.0%)	21 (12.5%)	10 (18.5%)	0.27
Asthma exacerbation	17 (7.7%)	16 (9.5%)	1 (1.9%)	0.08
Trauma	8 (3.6%)	5 (3.0%)	3 (5.6%)	0.41
Post cardiac arrest	11 (5.0%)	6 (3.6%)	5 (9.3%)	0.14
Post operative	13 (5.9%)	10 (6.0%)	3 (5.6%)	1.00

The cohort was stratified by in-hospital mortality at 30 days to analyse factors associated with survival (Table 1), 168 were alive and 54 had died at 30 days (75.6% survival rate). Descriptive differences between groups found those who survived were younger (63.9 vs. 71.5 years) than those who died. Overall, those who survived had a fewer preadmission comorbid burden with a lower Charlson Comorbidity Index (CCI) of 3 (IQR 1–4) compared to 5 (IQR 3–6) in those who died. Patients who died where more likely to have a diagnosis of COPD, bronchiectasis, hypertension or cancer within the last 5 years and/or to be on immunosuppressive medications. Asthma diagnosis had a better survival rate than COPD (Table 1).

### Respiratory support, other organ supportive measures and other ICU outcomes

**Fig. 1:**
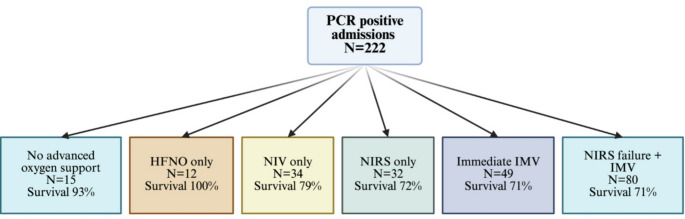
Details of respiratory support and 30-day mortality. HFNO: high flow nasal oxygen; IMV: invasive mechanical ventilation (IMV); NIV: non-invasive ventilation (including continuous positive airway pressure); NIRS: non-invasive respiratory support (any)

Among the 222 viral PCR-positive patients, only 15 patients (6.7%) were managed without advanced non-invasive or invasive respiratory support. 22.0% of patients received immediate mechanical ventilation without the trial of any NIRS measures and a further 36.0% received invasive mechanical ventilation after failing a ≥ 2 h trial of NRIS. The 30-day mortality was similar between patients who received immediate IMV or following NRIS failure (71%) (Fig. 1).

Across the whole cohort, cardiovascular support and renal replacement therapy was needed for 60.4% and 18.6% respectively. Cardiovascular support was much more common (81.5%) in those who died than survived (Table 2). Those who had mechanical ventilation, the median duration of mechanical ventilation was 7.0 (4.0,14.0) days. ICU and hospital length of stay were 14.5 (9.0, 28.0) and 17.0 (9.0, 34.5) days respectively. For those who died, there was a median ICU length of stay of 13.0 (6.0, 20.5) days with 66.7% dying in ICU (Table 2).

**Table 2:** Other organ support measures and outcomes for patients with severe viral infections admitted to intensive care. ICU, intensive care unit; IMV, invasive mechanical ventilation; HFNO, high flow nasal oxygen; NIRS, Non-invasive respiratory support; NIV, Non-invasive ventilation; RRT, renal replacement therapy. ^1^ Mann-Whiney U test and fischer’s exact test

	All	Survived *N* = 168	Died *N* = 54	*p*-value^1^
**Other organ support**				
Cardiovascular support, n (%)	134 (60.4%)	90 (53.6%)	44 (81.5%)	**< 0.01**
RRT therapy, n (%)	41 (18.6%)	28 (16.7%)	13 (24.5%)	0.23
**Other Outcomes**				
ICU length of stay (days)	14.5 (9.0–28.0)	15.5 (9.0-31.5)	13.0 (6.0-20.5)	**0.03**
Hospital length of stay (days)	17.0 (9.0-34.5)	17.0 (9.3–36.8)	15.5 (7.8–29.0)	0.22
Length of IMV (days)	7.0 (4.0, 14.0)	7.0 (4.0, 15.0)	8.0 (3.0, 11.0)	0.51

### Viral aetiology and other positive microbiology

**Fig. 2:**
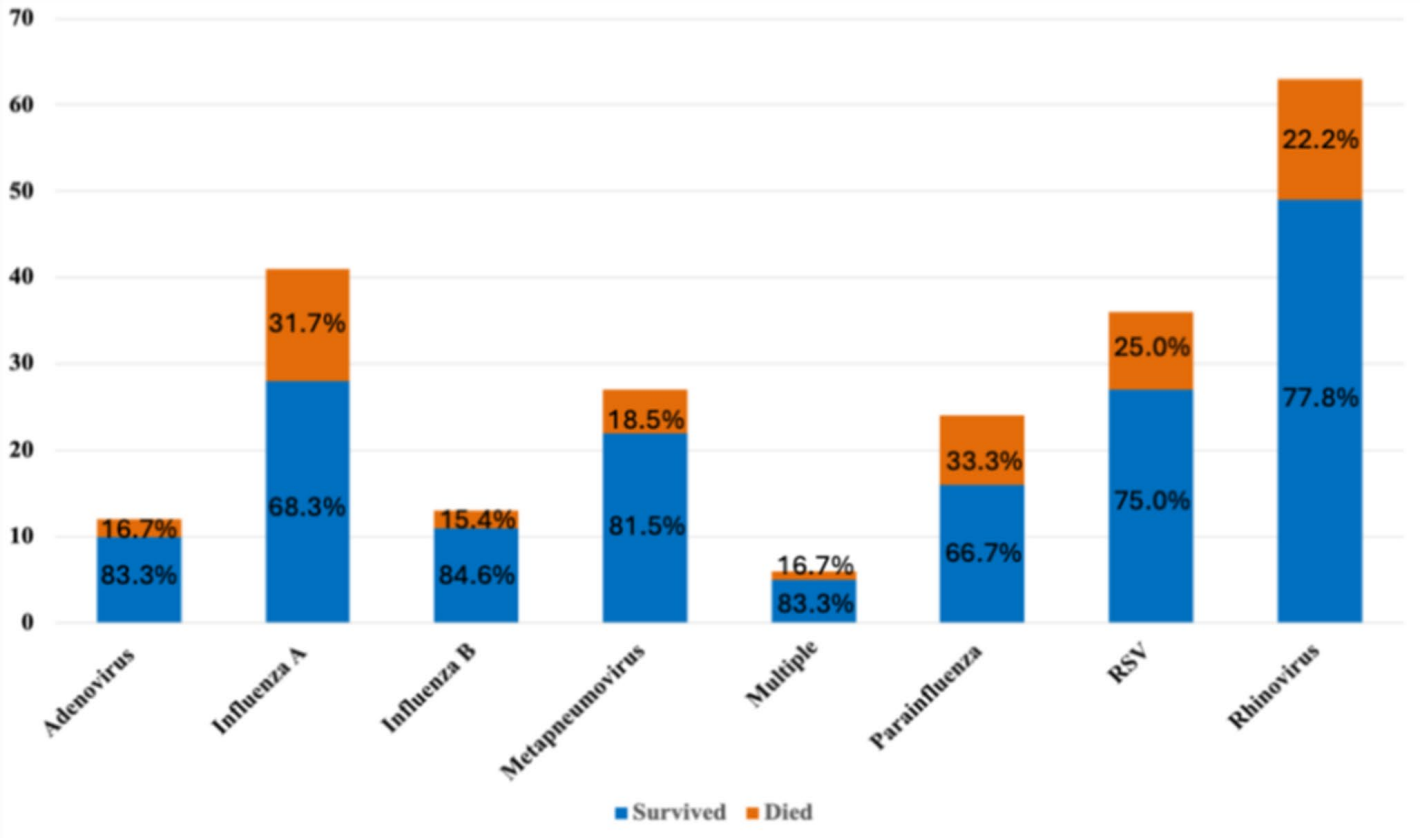
The number of patients with individual viral infections stratified according to in hospital 30-day mortality. The individual bars consist of the percentage of patients survived and died for each viral aetiology

Viral PCR testing revealed that patients were most frequently infected with rhinovirus (28.0%), influenza A (18.5%), respiratory syncytial virus (RSV) (16.2%), metapneumovirus (12.2%), parainfluenza (10.8%), influenza B (5.9%), adenovirus (5.4%), and a minority had multiple species, where more than one pathogen was detected (2.7%). Figure 2 depicts the viral aetiology stratified according to in-hospital 30-day mortality.

**Fig. 3:**
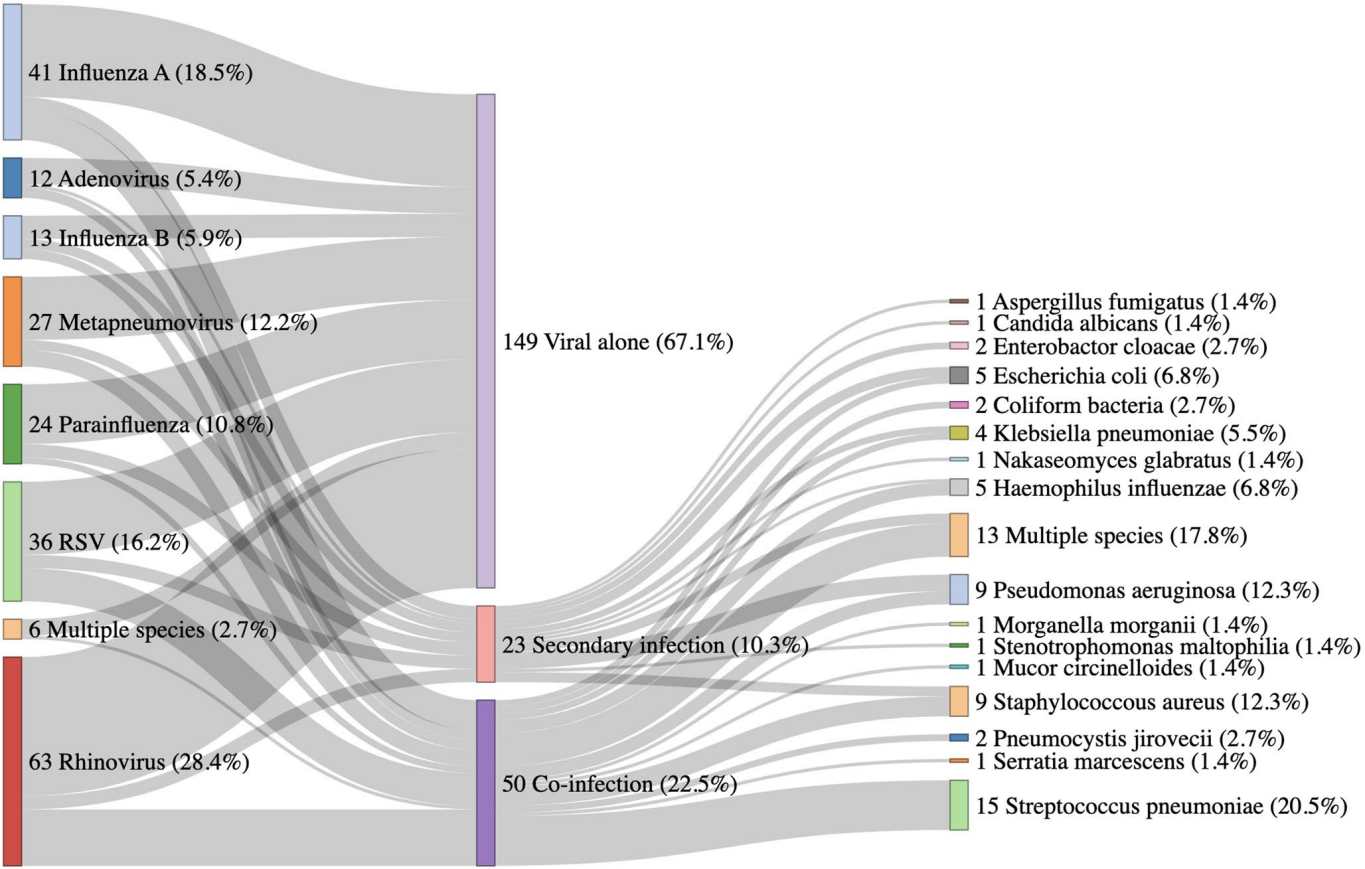
Sankey diagram describing the aetiology of severe viral infections and clinically important microbiological species for patients. Results on the left relate to viral PCR results, results on right relate to microbiological results, central stratification by viral alone, secondary infection and co-infection

Seventy-three patients (32.8%) with positive viral PCR returned other clinically significant microbiological results from a combination of blood cultures, respiratory tract fluids (tracheal aspirates or bronchoalveolar lavage), or urine antigen for pneumococcal and legionella on or within seven days from their ICU admission (Supplementary table S2). Of the 222 with detectable viral PCR results, 149 had no other positive microbiology, 50 had microbiologically confirmed co-infection (other positive microbiology within 48 h of presentation), and 23 developed secondary infections (microbiological sample > 48 h after presentation) (Fig. 3). While *Candida spp* were detected in seven patients, we regarded this as clinically relevant in two patients (*C. albicans* and *C. glabrata* respectively) who were immunosuppressed and were treated with antifungals. Immunosuppressed patients also had other isolates- one patient with *Aspergillus fumigatus*, two with *Pneumocystis jirovecii and one with Mucor circinelloides* secondary infections (Fig. 3).

Patients with pneumonia were more likely to have influenza A (82.9%), or adenovirus (83.3%) than rhinovirus (66.7%) (Supplementary table S3). Viral aetiology was similar between viral, co-infection, and secondary infection (Supplementary table S4). In patients with clinically significant microbiological samples (Supplementary table S5), *Streptococcus pneumoniae* (30.0%) was the most common pathogen found in co-infections, whilst *Pseudomonas spp*. (21.7%) was most common secondary infections treated, and *Staphylococcus aureus* and multiple species were detected in both co-infections and secondary infections.

### Routine blood markers

Routine blood markers at the point of ICU admission were analysed, these included CRP, neutrophil and lymphocyte count and platelet counts. There were differences between patients with viral infection alone, co-infection and secondary infection (Table 3). White cell count, lymphocytes, neutrophils, lymphocyte-to-neutrophil ratio and C reactive protein were similar between all groups for the first week of admission (Figure S1). Discernible differences were evident in platelet counts, with lower counts observed in co-infection and the lowest in secondary infection compared to patients with viral infection alone, reflecting the possibility of consumptive pathology due to immunothrombosis.

**Table 3:** Table of routine blood markers at the point of admission to the intensive care unit. Abbreviation: CRP, C reactive protein; WCC, white cell count

Variable at admission	Viral alone *N* = 149		Co-infection *N* = 50	Secondary infection *N* = 23	*p*-value
WCC		11.6 (7.7, 16.8)	11.5 (6.6, 13.9)	13.5 (8.1, 16.9)	0.52
Lymphocytes		0.7 (0.4, 1.2)	0.6 (0.4, 1.1)	0.7 (0.2, 1.0)	0.57
Neutrophils		9.7 (6.4, 13.7)	9.4 (5.8, 12.5)	10.7 (6.1, 14.5)	0.73
Neutrophil -lymphocyte ratio		13.8 (8.8, 25.0)	13.4 (8.5, 21.8)	15.1 (9.3, 30.2)	0.63
Platelets		239.8 (171.5, 339.9)	210.3 (130.3, 283.3)	196.0 (94.5, 279.3)	**0.04**
CRP		129.0 (50.0, 225.0)	151.5 (101.5, 220.0)	109.0 (45.0, 215.0)	0.19

### Clinical comparison of patients with viral and Microbiological infections

Demographics and admission were compared between viral alone, co infection and patients with secondary infection (Supplementary table S6). Those with co-infections and secondary infections were more frequently male and of lower BMI than those with viral infection alone. Charlson Comorbidity index was 4.0 (IQR 3.0–5.0) for secondary infections, 3.0 (IQR 1.0–4.0) for co infection, and 3.0 (IQR 1.0–5.0) for viral infection alone. Reasons for admission were similar between groups. Despite having only viral PCR positive and no other positive microbiology (*N* = 149), 88% of viral alone patients received antibiotics.

More patients were mechanically ventilated and required cardiovascular support in both co-infection and secondary infection groups. While the 30-day in hospital mortality was similar between groups, there was increased duration of ICU and hospital length of stay in those who had secondary infections. The distribution of other modalities of respiratory support were similar between groups. While these results suggest that patients with co-infection and who developed secondary infections may be sicker than the patients with viral infection alone, the overall 30-day mortality was similar between groups (Table 4).

**Table 4:** Respiratory support and outcomes for patients with severe viral infections admitted to intensive care. ^1^ comparison between viral alone and Co infection. ^2^ comparison between viral alone and secondary infection. Abbreviation: ICU, intensive care unit; IMV, invasive mechanical ventilation; HFNO, high flow nasal oxygen; NIRS, Non-invasive respiratory support; NIV, Non-invasive ventilation

	Viral alone *N* = 149	Co infection *N* = 50	Secondary infection *N* = 23	*P* value^1^	*P* value^2^
**Respiratory support**					
None required	13 (8.7%)	1 (2.0%)	1 (4.3%)	0.20	0.70
HFNO alone	12 (8.1%)	0 (0.0%)	0 (0.0%)	0.04	0.37
NIV alone	23 (15.4%)	9 (18.0%)	2 (8.7%)	0.67	0.54
NIRS alone	26 (17.4%)	4 (8.0%)	2 (8.7%)	0.11	0.38
Immediate IMV	28 (18.8%)	13 (26.0%)	8 (34.8%)	0.28	0.10
NIRS + IMV	47 (31.5%)	23 (46.0%)	10 (43.5%)	0.06	0.26
IMV	75 (50.3%)	36 (72.0%)	18 (78.3%)	**0.01**	**0.01**
**Other organ support**					
Cardiovascular	79 (53.0%)	35 (70.0%)	20 (87.0%)	**0.04**	**< 0.01**
Renal replacement therapy	26 (17.6%)	10 (20.0%)	5 (21.7%)	0.70	0.57
**Outcomes**					
30-day mortality	38 (25.5%)	11 (22.0%)	5 (21.7%)	0.62	0.70
ICU mortality	26 (17.4%)	7 (14.0%)	5 (21.7%)	0.57	0.57
ICU length of stay	13.0 (8.0–25.0)	17.0 (8.5–22.0)	31.0 (17.5–45.3)	0.80	**< 0.01**
Hospital length of stay	15.0 (9.0–29.0)	18.0 (9.5–35.0)	32.5 (25.8–47.3)	0.43	**< 0.01**
Length of IMV	7.0 (3.8–15.0)	7.0 (3.8–11.3)	11.5 (5.3–19.0)	0.49	0.29

### Clinical comparisons of patients with pneumonia stratified according to the type of viral infection

Of those with only viral PCR positive and no other positive microbiology (*n* = 149), 68.5% had radiological evidence of pneumonia. We assessed the 30-day mortality, stratified according to the type of viral infection and if they had radiological evidence of pneumonia. This suggests that pneumonia was seen in all viral infections, and the 30-day mortality was comparable between those with radiological evidence of pneumonia (25.5%) and those without (25.5%) (Table 5).

**Table 5:** 30-day mortality and viral aetiology for patients with viral infection alone stratified by pneumonia and non-pneumonia. Abbreviation: PCR, polymerase chain reaction; RSV, respiratory syncytial virus

Viral PCR	Pneumonia *N* = 102		Non-pneumonia *N* = 47	
	Survived *N* = 76	Died *N* = 26	Survived *N* = 35	Died *N* = 12
Adenovirus	5 (83.3%)	1 (16.7%)	2 (100.0%)	0 (0.0%)
Influenza A	16 (75.0%)	6 (25.0%)	2 (33.3%)	4 (66.7%)
Influenza B	6 (100.0%)	0 (0.0%)	0 (0.0%)	1 (100.0%)
Metapneumovirus	10 (83.3%)	2 (16.7%)	5 (71.4%)	2 (28.6%)
Multiple species	1 (50.0%)	1 (50.0%)	3 (100.0%)	0 (0.0%)
Parainfluenza	8 (66.7%)	4 (33.3%)	4 (66.7%)	2 (33.3%)
Rhinovirus	17 (65.4%)	9 (34.6%)	14 (87.5%)	2 (12.5%)
RSV	13 (81.3%)	3 (18.7%)	5 (83.3%)	1 (16.7%)

### Survival analysis and confounders

Cox proportional hazards models were constructed to compare co-infection and secondary infection to those with viral alone for the event – death at any point during the hospital admission. Unadjusted analysis of co-infection found a hazard ratio (HR) of 0.60 (95% confidence interval (CI) 0.30–1.19; *P* = 0.14) and secondary infection 0.91 (95% CI 0.47–1.76; *P* = 0.8) compared to viral infection. After adjustment for Charlson Comorbidity Index and age, HR was 0.84 (95% CI 0.41–1.73; *P* = 0.6) and 1.02 (95% CI 0.52–1.99; *P* > 0.9) for co-infection and secondary infection respectively (Table 6).

**Table 6:** Survival models for unadjusted and adjusted for age and Charlson comorbidity index during hospital admission. HR, hazard ratio; CI, confidence interval

Characteristic	HR	95% CI	*p*-value
Unadjusted analysis			
Viral alone	—	—	
Co infection	0.60	0.30,1.19	0.14
Secondary infection	0.91	0.47, 1.76	0.78
Adjusted analysis			
Viral alone	—	—	
Co infection	0.84	0.41, 1.73	0.64
Secondary infection	1.02	0.52, 1.99	0.96
Age	1.00	0.98, 1.02	0.82
Charlson comorbidity score	1.23	1.08, 1.39	**< 0.01**

## Discussion

In this study of critically ill patients admitted to the ICU, 222 patients were identified with detectable viral PCR results during the study period. Among these patients, the in-hospital 30-day survival was 75.7%. Approximately 40% had chronic respiratory comorbidities, primarily COPD (20%) and asthma (18%). 73% had radiological evidence of pneumonia. The most common viral infection identified was rhinovirus (28.4%), followed by Influenza A (18.5%) and RSV (16.2%). Clinical management of patients with severe viral infection remains challenging for ICUs with a high burden of respiratory support (93%), including non-invasive methods (CPAP/NIV/HFNO) or invasive mechanical ventilation. In turn, this is implicated in clinical outcomes, whereby the 30-day in-hospital survival rate for those who had invasive mechanical ventilation was 71%. Of the 22.5% of patients admitted with co-infections, the most common being *Streptococcus pneumoniae*, followed by *Staphylococcus aureus*. Some patients (10.4%) developed secondary infections defined as new positive microbiology after 48 h of ICU admission. Although patients with secondary infections had a prolonged ICU and hospital stay, the consequence of a subsequent microbiological infection did not translate to mortality, with 30-day in-hospital mortality being similar between these three groups. To the best of our knowledge, this study represents the largest dataset detailing severe respiratory viral infections in critically ill patients within the ICU, providing detailed information on specific ICU respiratory interventions and outcomes based on associated microbial infections.

Viral pathogens are identified in around 22–27% of hospitalised patients with pneumonia, and the commonly identified viruses include rhinovirus and influenza [10, 11, 21]. Incidence of viral infections in the intensive care unit patients with pneumonia is also relatively common ranging between 16 and 49% [8]. In a study of 229 ICU patients admitted with either community-acquired, or health-care-associated severe CAP (sCAP), 36.4% had positive viral PCR and 9.1% had viral-bacterial co-infections [6]. Moreover, similar to our findings, rhinovirus was the most prevalent virus (23.6%). Similarly, in another study of all consecutive mechanically ventilated patients, where 22% of patients had detectable virus by PCR, the most frequently detected virus was rhinovirus [22]. Despite being commonly considered a less pathogenic virus, commonly associated with a self-limiting upper respiratory tract infection, our study confirms previous findings that rhinovirus plays a dominant role in lower respiratory tract infections including pneumonia [23] and can produce severe pneumonia necessitating critical care. The risk factors for positive viral PCR includes admission with respiratory disorder, asthma/COPD and admission during the winter endemic season [22]. Similarly, in our cohort 40% had chronic respiratory conditions including COPD and asthma, reflecting increased tendency for viral infections in chronic respiratory conditions.

The use of non-invasive respiratory support for severe respiratory tract viral infections in the ICU population remains understudied. The recent COVID-19 pandemic highlighted the utility of non-invasive interventions (NIRS) such as HFNO, CPAP/NIV in patients with acute respiratory failure. Among the 158 patients who were treated with HFNO and or CPAP/NIV, 51% failed non-invasive respiratory intervention strategies and subsequently required invasive mechanical ventilation. A randomised controlled trial of COVID-19 patients treated with CPAP or HFNO found that 33% of those in the CPAP group and 40% of those in the HFNO group required subsequent mechanical ventilation and the use of CPAP was associated with a reduced need for mechanical ventilation and mortality at 30-days when compared to conventional oxygen therapy [24]. Whilst our patient group had a higher failure rate of around 50%, this is similar to a previous study on NIV use in patients with influenza [25]. In our unit, while patients are typically initiated on CPAP, most receive a combination of NIV and CPAP, with HFNO offered during breaks. Consequently, we were unable to distinguish between those treated with NIV and those with CPAP. Previously, we explored the use of NIV in unselected patients with sCAP, including patients with bacterial pneumonia, and identified a failure rate of 41% needing subsequent mechanical ventilation [26]. While NIV can be beneficial for some patients, the high failure rate necessitates careful close monitoring in a high dependency or ICU setting to mitigate risks related to delayed intubation.

Viral-bacterial or viral-other pathogens and polymicrobial severe respiratory tract co-infections are frequently observed in ICU settings. In our study, we found that 22.5% of patients with a positive viral PCR also had other positive microbiology within 48 h of admission. The most common bacteria identified was *Streptococcus pneumoniae* (30.0%), while the predominant viral infection associated with co-infection was rhinovirus (34.0%). We also assessed secondary infections in those admitted with a positive viral PCR. Twenty-three patients (10.4%) had additional positive non-viral microbiology after 48 h and within 7 days of ICU admission, which was deemed to be clinically significant by the intensive care infection specialist and received appropriate antimicrobial agents. The common isolate was *Pseudomonas aeruginosa* (21.7%), followed by *Escherichia coli* (13,0%), S*taphylococcus aureus* (13.0%) and multiple species (13.0%).

Viral infections can predispose to bacterial co-infections through several mechanisms, including the disruption of epithelial barriers, impaired mucociliary clearance, suppression of innate immune responses and increased expression of receptors that facilitate bacterial adhesion, which can result in synergistic pathogenesis resulting in poor outcomes [27–31] Studies investigating H1N1 influenza- bacterial co-infections have demonstrated that patients with such co-infections experience higher rates of mechanical ventilation, increased incidence of acute respiratory distress syndrome (ARDS) and higher mortality rates [32, 33]. Moreover, a systematic review of COVID-19 patients suggests that while the co-infection rate was relatively low- around 14% in the ICU setting, this was associated with worse clinical outcomes [34]. However, our study did not find any difference in mortality between viral infection, co-infection and secondary infection groups. Several factors could explain this finding: (1) differences in patient population- our study focused exclusively on critically ill patients in the ICU, and once the patient is in an ICU setting, outcomes may be similar regardless of the microbial status (ceiling of severity), (2) variations in pathogenic virulence between viruses- our study did not include COVID-19 patients where there is strong evidence that SARS-CoV-2 impairs immune defences promoting secondary bacterial infections and (3) 90% our patients received antibiotics on admission and timely administration of empiric broad-spectrum antibiotics may have mitigated the negative impact of co-infection or antimicrobial capture in other groups. However, similar to our findings, a previous study of unselected viral aetiology in the ICU population also reported no mortality difference between viral infections and viral-bacterial co-infections [6].

There are several limitations to our study. First, this retrospective single-centre study only evaluated patients with positive viral PCR results, excluding those with a clinical suspicion of infection who did not have positive PCR results or those who did not undergo PCR analysis. Second, the collection of respiratory samples was not uniform, as it included different methods such as BALF, tracheal aspirates, upper respiratory tract nasopharyngeal swabs and sputum traps, each of which may have different sensitivity and microbiological detection rates, which may have varying PCR sensitivity and microbiological capture. Third, we only included patients admitted to the ICU, which limits the study’s generalisability to less severe cases outside the ICU and highlights a potential source of bias around case identification. Fourth, it was also challenging to differentiate the dominant pathology (viral or other microbiological) in some cases that led to ICU admission, highlighting common issues in real-world practice. Fifth, this is a single centre study, and the clinical practices may differ internationally and may not be generalisable, particularly clinical practices may have been influenced by the recent COVID-19 pandemic. Lastly, we did not account for the prior antibiotics use before ICU admission, or precise details of type and duration of antibiotic therapy, which may have affected our microbiological detection rate, and subsequent bias towards the null effect. Despite these limitations, our findings remain highly clinically relavent as they represent a comprehensive analysis of a real-world cohort. Our findings are consistent with previous data on ICU populations and as far as we are aware, this is the largest ICU cohort of severe respiratory tract infections detailing co-infections and secondary infections with the requirement of organ support measures reported to date, adding the valuable clinical data to this evolving area.

## Conclusions

In this study, we provide a detailed analysis of a large cohort of critically ill patients with detectable respiratory viral infections over a 9-year period. The most commonly detected virus was rhinovirus. Nearly quarter of patients had a co-infection during the first 48 h of their admission, the commonest bacterial pathogen being *Streptococcus pneumoniae*. Secondary infections (after 48 h and within 7 days) occurred in 10% of patients. Most patients required advanced respiratory support and nearly 60% was invasively ventilated. Although there was an increased ICU and hospital stay noted in patients with secondary infections, the 30-day mortality was similar between all groups. Severe respiratory viral infections, whether they occur alone or in combination with co-infections, continue to impose high morbidity and mortality (~ 25%). Our findings provide real-world data that help define the implications of several respiratory viral infections and highlight the potential impact on appropriate service planning, as well as the need for future interventional trials to evaluate these complex interactions further.

## Supplementary Information

Below is the link to the electronic supplementary material.

Supplementary Material 1

## Data Availability

Data will be available upon resealable request to the corresponding author.
