# Supplementary material for: Severe viral infections requiring intensive care unit admissions- aetiology, co-infections, respiratory interventions and outcomes
Source: Infection. 2025 Sep 9;54(1):143–54. doi: 10.1007/s15010-025-02637-2 (PMC12864227; doi:10.1007/s15010-025-02637-2)

# Supplementary material: Severe viral infections requiring intensive care unit admissions- aetiology, co-infections, respiratory interventions and outcomes

Brown M^1^, Abeer F^1^, Roe T^1^, Beecham R^1^, Arscott O^1^, Eastwood B^1^, Mahar S^1^, Montague M^1^, Neseam D^1^, Patel P^1^, Srinivasa J^1^, Greenwell A^1^, Thomas K^1^, Browning D^2^_,_ Wilson-Davies E^3^, Conway Morris A^4^, Grocott MPW^1,2,5,6^, Saeed K^1,2,5^, Dushianthan A^1,2,5,6^

1. General Intensive Care Unit, University Hospital Southampton NHS Foundation Trust, Tremona Road, Southampton, SO16 6YD, UK.
2. Microbiology Department, University Hospital Southampton NHS Foundation Trust, Tremona Road, Southampton, SO16 6YD, UK.
3. Southampton Specialist Virology Centre, University Hospital Southampton NHS Foundation Trust, Tremona Road, Southampton, SO16 6YD, UK.
4. University Division of Anaesthesia, Department of Medicine, Addenbrooke’s Hospital, University of Cambridge, Hills Road, Cambridge, CB2 0QQ, UK.
5. Integrative Physiology and Critical Illness Group, Clinical and Experimental Sciences, Faculty of Medicine, University of Southampton, Southampton, SO16 6YD, UK.
6. Perioperative and Critical Care theme, NIHR Southampton Biomedical Research Centre, University Hospital Southampton / University of Southampton, Southampton, SO16 6YD, UK.

## Corresponding Author

Associate Professor A Dushianthan

General Intensive care Unit

University Hospital Southampton NHS Foundation Trust

Tremona Road, Southampton, SO16 6YD

Phone: 07903943418

Email: [a.dushianthan@soton.ac.uk](mailto:a.dushianthan@soton.ac.uk)

#

# **Table S1**: Reasons for admission for patients with pneumonia and without pneumonia.

| **Reasons for admission** | Pneumonia  N = 162 | Non-pneumonia  N = 60 |
| --- | --- | --- |
| Multi-organ dysfunction | 54 (33.3%) | 17 (28.3%) |
| COPD exacerbation | 18 (11.1%) | 13 (21.7%) |
| Asthma exacerbation | 9 (5.6%) | 8 (13.3%) |
| Trauma | 5 (3.1%) | 3 (5.0%) |
| Post cardiac arrest | 3 (1.9%) | 8 (13.3%) |
| Post operative | 8 (4.9%) | 5 (8.3%) |
| Table of reasons for admission for those listed as penumonia versus those without pneumonia. Abbreviation: COPD, chronic obstructive pulmonary disease. | | |

# **Table S2:** Table of positive microbiological results for co-infection and secondary infection groups.

| **Sample source** | Co-infection  N = 50 | Secondary infection  N = 23 |
| --- | --- | --- |
| Bronchoalveolar lavage | 6 | 4 |
| Blood culture | 3 | 0 |
| Endotracheal aspirate | 14 | 12 |
| Sputum | 28 | 11 |
| Urinary antigen | 13 | 0 |
| Table of positive microbiological results for patients with co-infection and secondary infection. Sources are not mutually exclusive. | | |

# **Table S3**: Isolated organisms of patients for admissions with pneumonia and without pneumonia.

| **Viral PCR** | Pneumonia  N = 162 | Non-pneumonia  N = 60 |
| --- | --- | --- |
| Adenovirus | 10 (6.2%) | 2 (3.3%) |
| Influenza A | 34 (21.0%) | 7 (11.7%) |
| Influenza B | 11 (6.8%) | 2 (3.3%) |
| Metapneumovirus | 19 (11.7%) | 8 (13.3%) |
| Multiple | 3 (1.9%) | 3 (5.0%) |
| Parainfluenza | 17 (10.5%) | 7 (11.7%) |
| Rhinovirus | 42 (25.9%) | 21 (35.0%) |
| RSV | 26 (16.0%) | 10 (16.7%) |
| Table of positive viral polymerase chain reaction testing for patient with severe viral infection admitted to intensive care. Abbreviation: PCR, Polymerase chain reaction; RSV, Respratory syncytial virus. | | |

# Table S4: Isolated organisms within patients with viral alone, co-infection and secondary infection.

| **Viral PCR** | Overall  N = 222 | Viral alone  N = 149 | Co-infection  N = 50 | Secondary infection  N = 23 |
| --- | --- | --- | --- | --- |
| Adenovirus | 12 (5.4%) | 8 (5.4%) | 3 (6.0%) | 1 (4.3%) |
| Influenza A | 41 (18.5%) | 28 (18.8%) | 9 (18.0%) | 4 (17.4%) |
| Influenza B | 13 (5.9%) | 7 (4.7%) | 3 (6.0%) | 3 (13.0%) |
| Metapneumovirus | 27 (12.2%) | 19 (12.8%) | 5 (10.0%) | 3 (13.0%) |
| Multiple | 6 (2.7%) | 5 (3.4%) | 1 (2.0%) | 0 (0.0%) |
| Parainfluenza | 24 (10.8%) | 18 (12.1%) | 2 (4.0%) | 4 (17.4%) |
| Rhinovirus | 63 (28.4%) | 42 (28.2%) | 17 (34.0%) | 4 (17.4%) |
| RSV | 36 (16.2%) | 22 (14.8%) | 10 (20.0%) | 4 (17.4%) |
| Table of positive viral polymerase chain reaction testing for patient with severe viral infection admitted to intensive care. Abbreviation: PCR, Polymerase hain reaction; RSV, Respratory syncytial virus. | | | | |

#

# **Table S5**: Microbiological species isolated between viral alone, co-infection and secondary infection.

| **Microbiological species** | Overall  N = 73 | Co-infection  N = 50 | Secondary infection  N = 23 |
| --- | --- | --- | --- |
| *Aspergillus fumigatus* | 1 (1.4%) | 0 (0.0%) | 1 (4.3%) |
| *Candida albicans* | 1 (1.4%) | 0 (0.0%) | 1 (4.3%) |
| *Nakaseomyces glabratus* | 1 (1.4%) | 0 (0.0%) | 1 (4.3%) |
| *Coliform bacteria* | 2 (2.7%) | 2 (4.0%) | 0 (0.0%) |
| *Enterobactor cloacae* | 2 (2.7%) | 0 (0.0%) | 2 (8.7%) |
| *Escherichia coli* | 5 (6.8%) | 2 (4.0%) | 3 (13.0%) |
| *Haemophilus influenzae* | 5 (6.8%) | 4 (8.0%) | 1 (4.3%) |
| *Klebsiella pneumoniae* | 4 (5.5%) | 2 (4.0%) | 2 (8.7%) |
| *Multiple species* | 13 (17.8%) | 10 (20.0%) | 3 (13.0%) |
| *Morganella morganii* | 1 (1.4%) | 1 (2.0%) | 0 (0.0%) |
| *Mucor circinelloides* | 1 (1.4%) | 1 (2.0%) | 0 (0.0%) |
| *Pneumocystis jirovecii* | 2 (2.7%) | 2 (4.0%) | 0 (0.0%) |
| *Pseudomonas aeruginosa* | 9 (12.3%) | 4 (8.0%) | 5 (21.7%) |
| *Serratia marcescens* | 1 (1.4%) | 1 (2.0%) | 0 (0.0%) |
| *Staphylococcous aureus* | 9 (12%) | 6 (12.0%) | 3 (13.0%) |
| *Stenotrophomonas maltophilia* | 1 (1.4%) | 0 (0.0%) | 1 (4.3%) |
| *Streptococcus pneumoniae* | 15 (20.5%) | 15 (30.0%) | 0 (0%) |

# Table S6: Baseline characteristics between viral alone, co-infection and secondary bacterial infection.

| **Characteristics** | **Viral alone** | **Co-infection** | **Secondary infection** | **p-value** |
| --- | --- | --- | --- | --- |
| Number of patients | 149 | 50 | 23 |  |
| Age | 67.0 (52.9, 75.9) | 62.0 (48.6, 73.6) | 66.0 (49.4, 75.0) | 0.56 |
| Male | 70 (47.0%) | 37 (74.0%) | 14 (60.9%) | **< 0.01** |
| Ethnicity |  |  |  | 0.06 |
| Asian or Asian | 7 (4.7%) | 1 (2.0%) | 0 (0.0%) |  |
| Black or black | 1 (0.7%) | 1 (2.0%) | 0 (0.0%) |  |
| White | 124 (83.2%) | 37 (74.0%) | 21 (91.3%) |  |
| Other ethnicity | 0 (0.0%) | 3 (6.0%) | 0 (0.0%) |  |
| Unknown | 17 (11.4%) | 8 (16.0%) | 2 (8.7%) |  |
| BMI | 27.7 (23.4, 32.3) | 25.7 (23.0, 30.6) | 25.2 (22.2, 28.1) | **0.04** |
| **Comorbidities** |  |  |  |  |
| Charlson comorbidity index | 3.0 (1.0, 5.0) | 3.0 (1.0, 4.0) | 4.0 (3.0, 5.0) | 0.21 |
| Chronic respiratory condition | 68 (45.6%) | 17 (34.0%) | 7 (30.4%) | 0.19 |
| Asthma | 31 (20.8%) | 9 (18.0%) | 1 (4.3%) | 0.17 |
| COPD | 32 (21.5%) | 8 (16.0%) | 4 (17.4%) | 0.75 |
| Bronchiectasis | 2 (1.3%) | 1 (2.0%) | 1 (4.3%) | 0.39 |
| Interstitial lung disease | 4 (2.7%) | 2 (4.0%) | 0 (0.0%) | 0.82 |
| IHD | 17 (11.4%) | 4 (8.0%) | 1 (4.3%) | 0.65 |
| HTN | 51 (34.2%) | 13 (26.0%) | 6 (26.1%) | 0.49 |
| MI | 12 (8.1%) | 2 (4.0%) | 1 (4.3%) | 0.70 |
| CKD | 19 (12.8%) | 1 (2.0%) | 1 (4.3%) | **0.04** |
| Liver cirrhosis | 2 (1.3%) | 2 (4.0%) | 4 (17.4%) | **< 0.01** |
| Cancer | 35 (23.5%) | 11 (22.0%) | 5 (21.7%) | 1.00 |
| Smoker | 48 (32.2%) | 14 (28.0%) | 7 (30.4%) | 0.90 |
| **Medications prior to admission** | | | | |
| Inhaled steroids | 29 (19.5%) | 10 (20.0%) | 3 (13.0%) | 0.80 |
| Immunosuppressive drugs | 18 (12.1%) | 6 (12.0%) | 3 (13.0%) | 1.00 |
| Oral steroids | 36 (24.2%) | 8 (16.0%) | 5 (21.7%) | 0.52 |
| **Reason for admission** |  |  |  |  |
| Pneumonia | 102 (68.5%) | 43 (86.0%) | 17 (73.9%) | 0.05 |
| Multi-organ dysfunction | 44 (29.5%) | 16 (32.0%) | 11 (47.8%) | 0.22 |
| COPD exacerbation | 23 (15.4%) | 5 (10.0%) | 3 (13.0%) | 0.69 |
| Asthma exacerbation | 14 (9.4%) | 2 (4.0%) | 1 (4.3%) | 0.53 |
| Trauma | 4 (2.7%) | 2 (4.0%) | 2 (8.7%) | 0.25 |
| Post cardiac arrest | 10 (6.7%) | 0 (0.0%) | 1 (4.3%) | 0.14 |
| Post operative | 8 (5.4%) | 3 (6.0%) | 2 (8.7%) | 0.74 |
| Table of Baseline characteristics of patients with severe viral infections admitted to intensive care with viral infections, bacterial co-infection or those with a secondary bacterial infection.  **Abbreviations** **BMI**, Body mass index; **COPD**, Chronic obstructive pulmonary disease; **IHD**, ischaemic heart disease. | | | | |

**Figure S1**: Routine blood markers were collected for patients for the first 5 days of admission, stratified by viral alone, co infection and secondary infection groups.


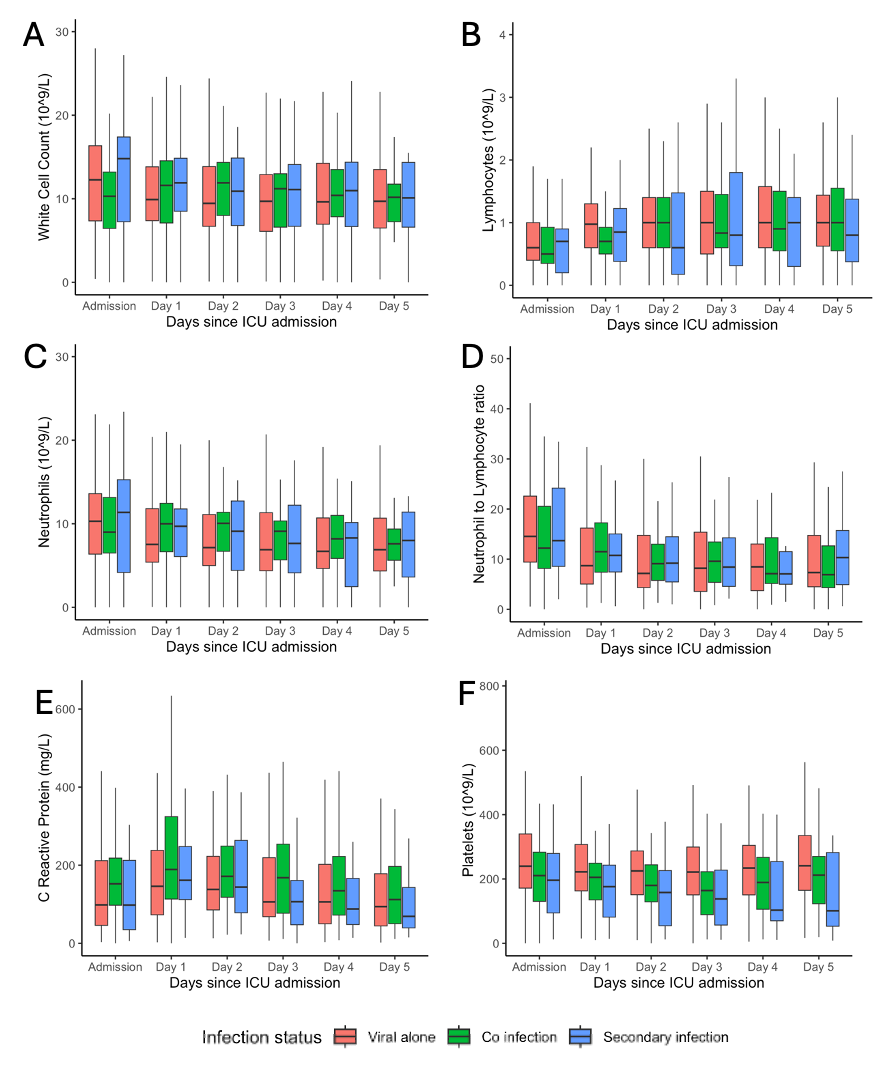

Supplement: Supplementary file 1 — Supplementary Material 1 [file 15010_2025_2637_MOESM1_ESM.docx]
